# New Appliances and Things Medical

**Published:** 1905-06-24

**Authors:** 


### NEW APPLIANCES AND THINGS MEDICAL.

[We shall be glad to receive at our Office, 28 & 29 Southampton Street, Strand, London, W.C., from the manufacturers, specimens of all new preparations and appliances which may be brought out from time to time.]

### LYSOFORM.

(THE BRITISH LYSOFORM COMPANY, LIMITED.)

WE have received a sample of this antiseptic, and the literature concerning it. The antiseptic power of formaldehyde is well known as is also that of formalin, which is a 40 per cent. solution of the above substance. An extraordinary property of this class of antiseptics is that while being in such extreme dilution inimical to low forms of life they are practically without poisonous effects upon the higher animals. Formic aldehyde and formalin possess, however, local irritant effects which render their use as such practically impossible. The preparation before us, lysoform, owes its great antiseptic power to its formic aldehyde moiety, it being a liquid formaldehyde potash soap. Its soapy nature is of immense advantage and gives it an almost unique position amongst antiseptics; when rubbed on the hands or skin it forms a lather and cleanses and disinfects at the same time. It is used as a basis for many toilet preparations, and is especially useful in dental work. We regard lysoform as quite one of the best antiseptics on the market, and upon reference to the original articles upon the subject it appears that this opinion is supported by many leading medical authorities.

### ANTIPHLOGISTINE.

(THE DENVER CHEMICAL MANUFACTURING COMPANY, DENVER, CHICAGO, AND NEW YORK.)

THIS somewhat explosive name is the title given to a new counter-irritant. The substance itself looks and feels remarkably like ordinary putty. This preparation, which is sent out in tins, must be gently warmed and applied with a brush, like paint, to the desired skin area. The painted area is then to be covered with strong linen or cotton. It can be left on 24 hours, and then with the linen easily peels off. It is antiseptic and anodyne, and by virtue of its affinity for water, and the superficial hyperæmia which it produces, it has a beneficial action in the absorption of local inflammatory exudations. It is recommended that in cases of pneumonia and bronchitis this application should replace the old-fashioned and still much used jacket poultices. All physicians admit the discomfort caused to the patient by the necessarily somewhat frequent changing of these poultices, but at the same time all are equally alive to the great relief generally afforded by their application. If the latter, as is claimed, can be obtained by the use of antiphlogistine, without the former, a great advance will have been made in this field of therapeutics.

